# (*E*)-1-(2,4-Dichloro­phen­yl)-3-(1,3-diphenyl-1*H*-pyrazol-4-yl)prop-2-en-1-one

**DOI:** 10.1107/S1600536811044382

**Published:** 2011-10-29

**Authors:** Hoong-Kun Fun, Ching Kheng Quah, Shridhar Malladi, Arun M. Isloor, Kammasandra N. Shivananda

**Affiliations:** a X-ray Crystallography Unit, School of Physics, Universiti Sains Malaysia, 11800 USM, Penang, Malaysia; b Medicinal Chemistry Division, Department of Chemistry, National Institute of Technology-Karnataka, Surathkal, Mangalore 575 025, India; c Schulich Faculty of Chemistry, Technion Israel Institute of Technology, Haifa 32000, Israel

## Abstract

In the title mol­ecule, C_24_H_16_Cl_2_N_2_O, the dihedral angles between the pyrazole ring and its N- and C-bonded phenyl rings are 7.06 (10) and 53.15 (10)°, respectively. The dihedral angle between the two pendant rings is 52.32 (10)°. The mol­ecule exists in a *trans* conformation with respect to the acyclic C=C bond. In the crystal, inversion dimers occur in which each mol­ecule is linked to the other by two C—H⋯O hydrogen bonds to the same acceptor O atom. There are also short Cl⋯Cl contacts [3.3492 (9) Å] and C—H⋯π inter­actions.

## Related literature

For general background to and the biological activity of pyrazoles, see: Patel *et al.* (2004 ▶); Isloor *et al.* (2009 ▶); Vijesh *et al.* (2010 ▶); Sharma *et al.* (2010 ▶); Rostom *et al.* (2003 ▶); Ghorab *et al.* (2010 ▶); Amnekar & Bhusari (2010 ▶). For hydrogen-bond motifs, see: Bernstein *et al.* (1995 ▶). For standard bond-length data, see: Allen *et al.* (1987 ▶).
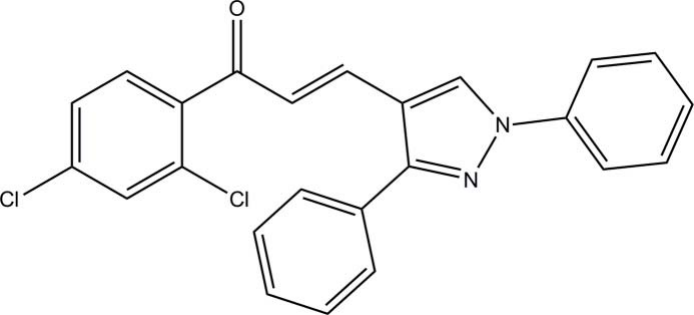


## Experimental

### 

#### Crystal data

C_24_H_16_Cl_2_N_2_O
*M* *_r_* = 419.29
Triclinic, 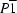

*a* = 9.6185 (8) Å
*b* = 10.6596 (9) Å
*c* = 11.8537 (10) Å
α = 67.377 (2)°
β = 75.777 (1)°
γ = 69.934 (2)°
*V* = 1044.64 (15) Å^3^
*Z* = 2
Mo *K*α radiation
μ = 0.33 mm^−1^
*T* = 296 K
0.31 × 0.21 × 0.08 mm

#### Data collection

Bruker SMART APEXII DUO CCD diffractometer
Absorption correction: multi-scan (*SADABS*; Bruker, 2009 ▶) *T*_min_ = 0.904, *T*_max_ = 0.973
22059 measured reflections
6053 independent reflections
3980 reflections with *I* > 2σ(*I*)
*R*_int_ = 0.027

#### Refinement

*R*[*F* ^2^ > 2σ(*F* ^2^)] = 0.043
*wR*(*F* ^2^) = 0.134
*S* = 1.04
6053 reflections
262 parameters
H-atom parameters constrained
Δρ_max_ = 0.22 e Å^−3^
Δρ_min_ = −0.27 e Å^−3^

### 

Data collection: *APEX2* (Bruker, 2009 ▶); cell refinement: *SAINT* (Bruker, 2009 ▶); data reduction: *SAINT*; program(s) used to solve structure: *SHELXTL* (Sheldrick, 2008 ▶); program(s) used to refine structure: *SHELXTL*; molecular graphics: *SHELXTL*; software used to prepare material for publication: *SHELXTL* and *PLATON* (Spek, 2009 ▶).

## Supplementary Material

Crystal structure: contains datablock(s) global, I. DOI: 10.1107/S1600536811044382/hb6456sup1.cif

Structure factors: contains datablock(s) I. DOI: 10.1107/S1600536811044382/hb6456Isup2.hkl

Supplementary material file. DOI: 10.1107/S1600536811044382/hb6456Isup3.cml

Additional supplementary materials:  crystallographic information; 3D view; checkCIF report

## Figures and Tables

**Table 1:** Hydrogen-bond geometry (Å, °) *Cg*1 and *Cg*2 are the centroids of the C19–C24 and C13–C18 benzene rings, respectively.

*D*—H⋯*A*	*D*—H	H⋯*A*	*D*⋯*A*	*D*—H⋯*A*
C11—H11*A*⋯O1^i^	0.93	2.30	3.230 (2)	174
C20—H20*A*⋯O1^i^	0.93	2.59	3.509 (3)	168
C2—H2*A*⋯*Cg*1^ii^	0.93	2.75	3.585 (2)	149
C23—H23*A*⋯*Cg*2^iii^	0.93	2.90	3.655 (2)	140

Symmetry codes: (i) 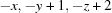; (ii) 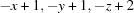; (iii) 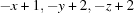.

**Fig. 1.**
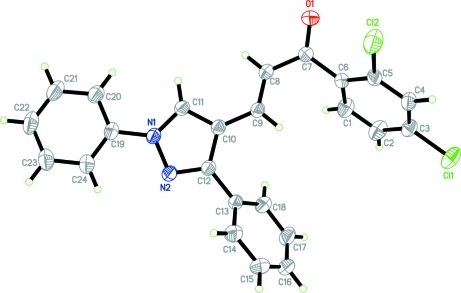
The molecular structure of the title compound showing 30% probability displacement ellipsoids for non-H atoms.

**Fig. 2.**
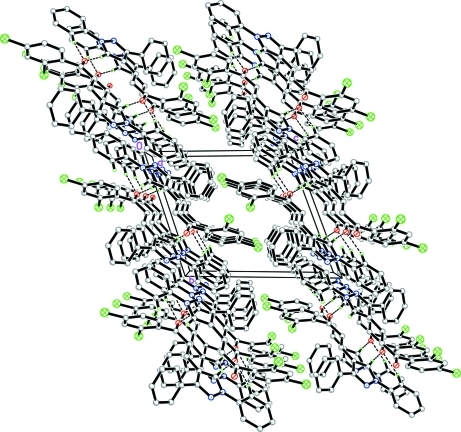
The crystal structure of the title compound, viewed along the a axis. H atoms not involved in hydrogen bonds (dashed lines) have been omitted for clarity.

## supplementary crystallographic information

### Comment

Pyrazoles are novel class of heterocyclic compounds possessing wide variety of application in the agrochemical and pharmaceutical industries (Patel *et al.*, 2004). Derivatives of pyrazoles are found to show good antibacterial (Isloor *et al.*, 2009; Vijesh *et al.*, 2010), anti-inflammatory (Sharma *et al.*, 2010), analgesic (Rostom *et al.*, 2003), anticancer, radioprotective (Ghorab *et al.*, 2010) and anti-convulsant activity (Amnekar & Bhusari, 2010). Prompted by the diverse activities of pyrazole derivatives, we have synthesized the title compound to study its crystal structure.

In the title molecule (Fig. 1), the phenyl (C1-C6) ring and the two benzene (C13-C18 and C19-C24) rings form dihedral angles of 64.29 (9), 53.15 (10) and 7.06 (10)°, respectively, with the pyrazole ring (N1/N2/C10-C12). The phenyl ring also forms dihedral angles of 65.06 (10) and 67.80 (10)° with the two benzene rings (C13-C18 and C19-C24), respectively. The benzene rings form a dihedral angle of 52.32 (10)°. The title molecule exists in *trans* conformation with respect to the acyclic C8═C9 bond [bond length = 1.330 (2) Å]. Bond lengths (Allen *et al.*, 1987) and angles are within normal ranges. There is a short Cl2···Cl2 contact (symmetry code : -x, 1-y, 1-z) with distance = 3.3492 (9) Å which is shorter than the sum of van der Waals radii of the Cl atoms.

In the crystal (Fig. 2), molecules are linked into inversion dimers by intermolecular bifurcated C11–H11A···O1 and C20–H20A···O1 acceptor bonds (Table 1), generating six-membered R^1^_2_(6) ring motifs (Bernstein *et al.*, 1995). The crystal structure is further consolidated by C2–H2A···Cg1 and C23–H23A···Cg2 (Table 1) interactions, where Cg1 and Cg2 are the centroids of C19-C24 and C13-C18 benzene rings, respectively.

### Experimental

To a cold, stirred mixture of methanol (20 ml) and sodium hydroxide (12.09 mmol), 2,4-dichloroacetophenone (4.03 mmol) was added. The reaction mixture was stirred for 10 min. 1,3-Diphenyl-1H-pyrazole-4-carbaldehyde (4.03 mmol) was added to this solution followed by tetrahydrofuran (30 ml). The solution was further stirred for 2 h at 273 K and then at room temperature for 5 h. It was then poured into ice cold water. The resulting solution was neutralized with Dil. HCl. The solid that separated was filtered, washed with water, dried and crystallized from ethanol to yield colourless blocks of (I). Yield: 1.28 g, 76.19 %. *M.p.*: 406-408 K.

### Refinement

All H atoms were positioned geometrically and refined using a riding model with C–H = 0.93 Å and *U*_iso_(H) = 1.2 *U*_eq_(C).

### Crystal data

**Table:**

C_24_H_16_Cl_2_N_2_O	*Z* = 2
*M**_r_* = 419.29	*F*(000) = 432
Triclinic, *P*1	*D*_x_ = 1.333 Mg m^−^^3^
Hall symbol: -P 1	Mo *K*α radiation, λ = 0.71073 Å
*a* = 9.6185 (8) Å	Cell parameters from 5364 reflections
*b* = 10.6596 (9) Å	θ = 2.4–28.8°
*c* = 11.8537 (10) Å	µ = 0.33 mm^−^^1^
α = 67.377 (2)°	*T* = 296 K
β = 75.777 (1)°	Block, colourless
γ = 69.934 (2)°	0.31 × 0.21 × 0.08 mm
*V* = 1044.64 (15) Å^3^	

### Data collection

**Table:**

Bruker SMART APEXII DUO CCD diffractometer	6053 independent reflections
Radiation source: fine-focus sealed tube	3980 reflections with *I* > 2σ(*I*)
graphite	*R*_int_ = 0.027
φ and ω scans	θ_max_ = 30.0°, θ_min_ = 1.9°
Absorption correction: multi-scan (*SADABS*; Bruker, 2009)	*h* = −13→13
*T*_min_ = 0.904, *T*_max_ = 0.973	*k* = −14→14
22059 measured reflections	*l* = −16→16

### Refinement

**Table:**

Refinement on *F*^2^	Primary atom site location: structure-invariant direct methods
Least-squares matrix: full	Secondary atom site location: difference Fourier map
*R*[*F*^2^ > 2σ(*F*^2^)] = 0.043	Hydrogen site location: inferred from neighbouring sites
*wR*(*F*^2^) = 0.134	H-atom parameters constrained
*S* = 1.04	*w* = 1/[σ^2^(*F*_o_^2^) + (0.0581*P*)^2^ + 0.1623*P*] where *P* = (*F*_o_^2^ + 2*F*_c_^2^)/3
6053 reflections	(Δ/σ)_max_ = 0.001
262 parameters	Δρ_max_ = 0.22 e Å^−^^3^
0 restraints	Δρ_min_ = −0.27 e Å^−^^3^

### Special details

**Table:**

Geometry. All esds (except the esd in the dihedral angle between two l.s. planes) are estimated using the full covariance matrix. The cell esds are taken into account individually in the estimation of esds in distances, angles and torsion angles; correlations between esds in cell parameters are only used when they are defined by crystal symmetry. An approximate (isotropic) treatment of cell esds is used for estimating esds involving l.s. planes.
Refinement. Refinement of F^2^ against ALL reflections. The weighted R-factor wR and goodness of fit S are based on F^2^, conventional R-factors R are based on F, with F set to zero for negative F^2^. The threshold expression of F^2^ > 2sigma(F^2^) is used only for calculating R-factors(gt) etc. and is not relevant to the choice of reflections for refinement. R-factors based on F^2^ are statistically about twice as large as those based on F, and R- factors based on ALL data will be even larger.

### Fractional atomic coordinates and isotropic or equivalent isotropic displacement parameters (Å^2^)

**Table:**

	*x*	*y*	*z*	*U*_iso_*/*U*_eq_	
Cl1	0.72422 (6)	0.23879 (6)	0.46250 (5)	0.08035 (18)	
Cl2	0.14184 (5)	0.45480 (9)	0.57917 (6)	0.1019 (3)	
O1	0.10827 (16)	0.35212 (16)	0.86276 (14)	0.0787 (4)	
N1	0.22996 (13)	0.84151 (14)	1.00579 (11)	0.0461 (3)	
N2	0.35901 (14)	0.86410 (14)	0.93127 (12)	0.0496 (3)	
C1	0.4853 (2)	0.3081 (2)	0.76848 (16)	0.0634 (5)	
H1A	0.5039	0.2962	0.8459	0.076*	
C2	0.6034 (2)	0.2677 (2)	0.68476 (17)	0.0677 (5)	
H2A	0.7000	0.2286	0.7056	0.081*	
C3	0.57655 (18)	0.28591 (17)	0.57036 (15)	0.0537 (4)	
C4	0.43478 (18)	0.34217 (18)	0.53839 (16)	0.0565 (4)	
H4A	0.4175	0.3541	0.4606	0.068*	
C5	0.31849 (17)	0.38071 (18)	0.62408 (16)	0.0526 (4)	
C6	0.33961 (17)	0.36581 (16)	0.74104 (14)	0.0470 (3)	
C7	0.21224 (19)	0.40361 (18)	0.83561 (15)	0.0536 (4)	
C8	0.21287 (19)	0.50151 (19)	0.89386 (15)	0.0570 (4)	
H8A	0.1421	0.5086	0.9621	0.068*	
C9	0.30613 (17)	0.58143 (16)	0.85751 (13)	0.0466 (3)	
H9A	0.3819	0.5680	0.7937	0.056*	
C10	0.30113 (16)	0.68689 (16)	0.90744 (13)	0.0452 (3)	
C11	0.19295 (17)	0.73726 (17)	0.99304 (14)	0.0481 (3)	
H11A	0.1098	0.7049	1.0343	0.058*	
C12	0.40123 (16)	0.77075 (16)	0.87221 (14)	0.0452 (3)	
C13	0.53890 (16)	0.76264 (17)	0.78363 (14)	0.0478 (3)	
C14	0.5637 (2)	0.8813 (2)	0.68817 (19)	0.0703 (5)	
H14A	0.4923	0.9684	0.6784	0.084*	
C15	0.6947 (3)	0.8706 (3)	0.6070 (2)	0.0862 (7)	
H15A	0.7098	0.9505	0.5420	0.103*	
C16	0.8019 (2)	0.7449 (3)	0.6209 (2)	0.0785 (6)	
H16A	0.8902	0.7395	0.5666	0.094*	
C17	0.7792 (2)	0.6270 (2)	0.71462 (19)	0.0711 (5)	
H17A	0.8523	0.5410	0.7244	0.085*	
C18	0.64774 (18)	0.6348 (2)	0.79530 (16)	0.0580 (4)	
H18A	0.6323	0.5535	0.8580	0.070*	
C19	0.15658 (16)	0.92254 (17)	1.08631 (14)	0.0488 (4)	
C20	0.03696 (19)	0.8896 (2)	1.17154 (17)	0.0612 (4)	
H20A	0.0011	0.8173	1.1752	0.073*	
C21	−0.0289 (2)	0.9666 (3)	1.25176 (19)	0.0756 (6)	
H21A	−0.1097	0.9455	1.3097	0.091*	
C22	0.0236 (2)	1.0730 (3)	1.2467 (2)	0.0799 (6)	
H22A	−0.0205	1.1228	1.3018	0.096*	
C23	0.1411 (2)	1.1064 (2)	1.1603 (2)	0.0755 (6)	
H23A	0.1757	1.1797	1.1561	0.091*	
C24	0.2084 (2)	1.03114 (19)	1.07925 (17)	0.0609 (4)	
H24A	0.2880	1.0538	1.0205	0.073*	

### Atomic displacement parameters (Å^2^)

**Table:**

	*U*^11^	*U*^22^	*U*^33^	*U*^12^	*U*^13^	*U*^23^
Cl1	0.0657 (3)	0.0801 (3)	0.0695 (3)	0.0003 (2)	0.0118 (2)	−0.0280 (3)
Cl2	0.0469 (3)	0.1805 (7)	0.1026 (4)	0.0004 (3)	−0.0210 (3)	−0.0934 (5)
O1	0.0746 (8)	0.1027 (10)	0.0849 (10)	−0.0569 (8)	0.0247 (7)	−0.0516 (8)
N1	0.0409 (6)	0.0555 (7)	0.0445 (7)	−0.0107 (5)	−0.0034 (5)	−0.0228 (6)
N2	0.0432 (6)	0.0584 (8)	0.0514 (7)	−0.0148 (6)	0.0002 (5)	−0.0258 (6)
C1	0.0607 (10)	0.0753 (11)	0.0465 (9)	−0.0052 (9)	−0.0152 (8)	−0.0190 (8)
C2	0.0498 (9)	0.0772 (12)	0.0580 (10)	0.0050 (8)	−0.0153 (8)	−0.0180 (9)
C3	0.0512 (8)	0.0471 (8)	0.0515 (9)	−0.0044 (7)	0.0001 (7)	−0.0165 (7)
C4	0.0537 (9)	0.0680 (10)	0.0519 (9)	−0.0095 (8)	−0.0069 (7)	−0.0306 (8)
C5	0.0435 (8)	0.0643 (10)	0.0600 (9)	−0.0117 (7)	−0.0080 (7)	−0.0330 (8)
C6	0.0491 (8)	0.0494 (8)	0.0472 (8)	−0.0172 (6)	−0.0011 (6)	−0.0210 (6)
C7	0.0549 (9)	0.0619 (9)	0.0507 (9)	−0.0251 (8)	0.0054 (7)	−0.0255 (7)
C8	0.0576 (9)	0.0704 (10)	0.0515 (9)	−0.0284 (8)	0.0132 (7)	−0.0321 (8)
C9	0.0467 (8)	0.0563 (9)	0.0390 (7)	−0.0165 (7)	0.0010 (6)	−0.0200 (6)
C10	0.0448 (7)	0.0514 (8)	0.0404 (7)	−0.0147 (6)	−0.0022 (6)	−0.0171 (6)
C11	0.0446 (7)	0.0584 (9)	0.0446 (8)	−0.0175 (7)	−0.0006 (6)	−0.0209 (7)
C12	0.0414 (7)	0.0522 (8)	0.0430 (8)	−0.0118 (6)	−0.0037 (6)	−0.0190 (6)
C13	0.0444 (8)	0.0591 (9)	0.0471 (8)	−0.0187 (7)	0.0000 (6)	−0.0250 (7)
C14	0.0740 (12)	0.0622 (11)	0.0730 (12)	−0.0283 (9)	0.0118 (10)	−0.0255 (9)
C15	0.0974 (16)	0.0867 (15)	0.0792 (14)	−0.0554 (13)	0.0292 (12)	−0.0311 (12)
C16	0.0657 (12)	0.1101 (17)	0.0835 (14)	−0.0461 (12)	0.0260 (10)	−0.0599 (14)
C17	0.0497 (9)	0.0921 (14)	0.0775 (13)	−0.0128 (9)	0.0048 (9)	−0.0482 (12)
C18	0.0497 (9)	0.0676 (10)	0.0536 (9)	−0.0132 (8)	−0.0020 (7)	−0.0223 (8)
C19	0.0430 (7)	0.0575 (9)	0.0451 (8)	−0.0012 (6)	−0.0116 (6)	−0.0238 (7)
C20	0.0487 (9)	0.0821 (12)	0.0594 (10)	−0.0145 (8)	−0.0031 (7)	−0.0360 (9)
C21	0.0540 (10)	0.1103 (17)	0.0666 (12)	−0.0113 (10)	0.0016 (9)	−0.0495 (12)
C22	0.0712 (12)	0.0989 (16)	0.0786 (14)	−0.0009 (11)	−0.0085 (11)	−0.0595 (13)
C23	0.0809 (13)	0.0743 (12)	0.0820 (14)	−0.0099 (10)	−0.0114 (11)	−0.0467 (11)
C24	0.0642 (10)	0.0610 (10)	0.0605 (10)	−0.0121 (8)	−0.0050 (8)	−0.0297 (8)

### Geometric parameters (Å, °)

**Table:**

Cl1—C3	1.7369 (16)	C11—H11A	0.9300
Cl2—C5	1.7304 (16)	C12—C13	1.474 (2)
O1—C7	1.2196 (19)	C13—C14	1.382 (2)
N1—C11	1.346 (2)	C13—C18	1.385 (2)
N1—N2	1.3718 (17)	C14—C15	1.385 (3)
N1—C19	1.4283 (19)	C14—H14A	0.9300
N2—C12	1.3282 (19)	C15—C16	1.360 (3)
C1—C2	1.378 (3)	C15—H15A	0.9300
C1—C6	1.388 (2)	C16—C17	1.362 (3)
C1—H1A	0.9300	C16—H16A	0.9300
C2—C3	1.370 (3)	C17—C18	1.384 (2)
C2—H2A	0.9300	C17—H17A	0.9300
C3—C4	1.371 (2)	C18—H18A	0.9300
C4—C5	1.378 (2)	C19—C24	1.377 (2)
C4—H4A	0.9300	C19—C20	1.379 (2)
C5—C6	1.391 (2)	C20—C21	1.388 (3)
C6—C7	1.501 (2)	C20—H20A	0.9300
C7—C8	1.458 (2)	C21—C22	1.368 (3)
C8—C9	1.330 (2)	C21—H21A	0.9300
C8—H8A	0.9300	C22—C23	1.371 (3)
C9—C10	1.441 (2)	C22—H22A	0.9300
C9—H9A	0.9300	C23—C24	1.384 (3)
C10—C11	1.385 (2)	C23—H23A	0.9300
C10—C12	1.416 (2)	C24—H24A	0.9300
			
C11—N1—N2	111.74 (12)	N2—C12—C13	120.29 (14)
C11—N1—C19	128.80 (13)	C10—C12—C13	127.91 (13)
N2—N1—C19	119.42 (13)	C14—C13—C18	118.39 (15)
C12—N2—N1	104.76 (12)	C14—C13—C12	121.32 (15)
C2—C1—C6	122.13 (16)	C18—C13—C12	120.28 (15)
C2—C1—H1A	118.9	C13—C14—C15	119.99 (19)
C6—C1—H1A	118.9	C13—C14—H14A	120.0
C3—C2—C1	119.06 (16)	C15—C14—H14A	120.0
C3—C2—H2A	120.5	C16—C15—C14	121.0 (2)
C1—C2—H2A	120.5	C16—C15—H15A	119.5
C2—C3—C4	121.28 (15)	C14—C15—H15A	119.5
C2—C3—Cl1	119.79 (13)	C15—C16—C17	119.70 (18)
C4—C3—Cl1	118.92 (13)	C15—C16—H16A	120.1
C3—C4—C5	118.53 (15)	C17—C16—H16A	120.1
C3—C4—H4A	120.7	C16—C17—C18	120.23 (19)
C5—C4—H4A	120.7	C16—C17—H17A	119.9
C4—C5—C6	122.56 (14)	C18—C17—H17A	119.9
C4—C5—Cl2	117.16 (12)	C17—C18—C13	120.67 (17)
C6—C5—Cl2	120.25 (12)	C17—C18—H18A	119.7
C1—C6—C5	116.43 (14)	C13—C18—H18A	119.7
C1—C6—C7	121.05 (14)	C24—C19—C20	120.82 (15)
C5—C6—C7	122.46 (14)	C24—C19—N1	119.36 (15)
O1—C7—C8	120.46 (15)	C20—C19—N1	119.81 (15)
O1—C7—C6	119.38 (15)	C19—C20—C21	118.66 (19)
C8—C7—C6	120.16 (14)	C19—C20—H20A	120.7
C9—C8—C7	125.46 (15)	C21—C20—H20A	120.7
C9—C8—H8A	117.3	C22—C21—C20	120.8 (2)
C7—C8—H8A	117.3	C22—C21—H21A	119.6
C8—C9—C10	126.31 (14)	C20—C21—H21A	119.6
C8—C9—H9A	116.8	C21—C22—C23	120.02 (19)
C10—C9—H9A	116.8	C21—C22—H22A	120.0
C11—C10—C12	104.10 (13)	C23—C22—H22A	120.0
C11—C10—C9	128.48 (14)	C22—C23—C24	120.2 (2)
C12—C10—C9	127.26 (13)	C22—C23—H23A	119.9
N1—C11—C10	107.60 (13)	C24—C23—H23A	119.9
N1—C11—H11A	126.2	C19—C24—C23	119.49 (18)
C10—C11—H11A	126.2	C19—C24—H24A	120.3
N2—C12—C10	111.80 (13)	C23—C24—H24A	120.3
			
C11—N1—N2—C12	−0.17 (16)	N1—N2—C12—C13	−179.30 (13)
C19—N1—N2—C12	177.93 (12)	C11—C10—C12—N2	0.29 (17)
C6—C1—C2—C3	0.5 (3)	C9—C10—C12—N2	175.95 (14)
C1—C2—C3—C4	−0.6 (3)	C11—C10—C12—C13	179.43 (15)
C1—C2—C3—Cl1	178.80 (15)	C9—C10—C12—C13	−4.9 (3)
C2—C3—C4—C5	0.1 (3)	N2—C12—C13—C14	−52.8 (2)
Cl1—C3—C4—C5	−179.29 (13)	C10—C12—C13—C14	128.08 (19)
C3—C4—C5—C6	0.5 (3)	N2—C12—C13—C18	126.52 (17)
C3—C4—C5—Cl2	178.65 (14)	C10—C12—C13—C18	−52.6 (2)
C2—C1—C6—C5	0.1 (3)	C18—C13—C14—C15	0.1 (3)
C2—C1—C6—C7	177.56 (17)	C12—C13—C14—C15	179.44 (18)
C4—C5—C6—C1	−0.7 (3)	C13—C14—C15—C16	−1.3 (4)
Cl2—C5—C6—C1	−178.70 (14)	C14—C15—C16—C17	1.2 (4)
C4—C5—C6—C7	−178.03 (16)	C15—C16—C17—C18	0.1 (3)
Cl2—C5—C6—C7	3.9 (2)	C16—C17—C18—C13	−1.3 (3)
C1—C6—C7—O1	−123.5 (2)	C14—C13—C18—C17	1.2 (3)
C5—C6—C7—O1	53.8 (2)	C12—C13—C18—C17	−178.17 (16)
C1—C6—C7—C8	56.8 (2)	C11—N1—C19—C24	−176.03 (15)
C5—C6—C7—C8	−125.95 (18)	N2—N1—C19—C24	6.2 (2)
O1—C7—C8—C9	−168.14 (18)	C11—N1—C19—C20	5.2 (2)
C6—C7—C8—C9	11.6 (3)	N2—N1—C19—C20	−172.51 (14)
C7—C8—C9—C10	174.67 (16)	C24—C19—C20—C21	−1.1 (3)
C8—C9—C10—C11	−7.0 (3)	N1—C19—C20—C21	177.64 (16)
C8—C9—C10—C12	178.37 (17)	C19—C20—C21—C22	0.0 (3)
N2—N1—C11—C10	0.35 (17)	C20—C21—C22—C23	1.0 (3)
C19—N1—C11—C10	−177.52 (14)	C21—C22—C23—C24	−1.0 (3)
C12—C10—C11—N1	−0.37 (16)	C20—C19—C24—C23	1.2 (3)
C9—C10—C11—N1	−175.96 (14)	N1—C19—C24—C23	−177.55 (16)
N1—N2—C12—C10	−0.08 (16)	C22—C23—C24—C19	−0.1 (3)

### Hydrogen-bond geometry (Å, °)

**Table:**

Cg1 and Cg2 are the centroids of the C19–C24 and C13–C18 benzene rings, respectively.

**Table:**

*D*—H···*A*	*D*—H	H···*A*	*D*···*A*	*D*—H···*A*
C11—H11A···O1^i^	0.93	2.30	3.230 (2)	174
C20—H20A···O1^i^	0.93	2.59	3.509 (3)	168
C2—H2A···Cg1^ii^	0.93	2.75	3.585 (2)	149
C23—H23A···Cg2^iii^	0.93	2.90	3.655 (2)	140

Symmetry codes: (i) −*x*, −*y*+1, −*z*+2; (ii) −*x*+1, −*y*+1, −*z*+2; (iii) −*x*+1, −*y*+2, −*z*+2.
